# Deletion of the Zinc Transporter Lipoprotein AdcAII Causes Hyperencapsulation of Streptococcus pneumoniae Associated with Distinct Alleles of the Type I Restriction-Modification System

**DOI:** 10.1128/mBio.00445-20

**Published:** 2020-03-31

**Authors:** Claire Durmort, Giuseppe Ercoli, Elisa Ramos-Sevillano, Suneeta Chimalapati, Richard D. Haigh, Megan De Ste Croix, Katherine Gould, Jason Hinds, Yann Guerardel, Thierry Vernet, Marco Oggioni, Jeremy S. Brown

**Affiliations:** a Institut de Biologie Structurale (IBS), Univ. Grenoble Alpes, CEA, CNRS, Grenoble, France; b Centre for Inflammation and Tissue Repair, Department of Medicine, Royal Free and University College Medical School, Rayne Institute, London, United Kingdom; c Department of Genetics and Genome Biology, University of Leicester, Leicester, United Kingdom; d Institute for Infection and Immunity, St. George’s University of London, London, United Kingdom; e Univ. Lille, CNRS, UMR 8576—UGSF-Unité de Glycobiologie Structurale et Fonctionnelle, Lille, France; University of Lausanne; University of Mississippi Medical Center

**Keywords:** *Streptococcus pneumoniae*, capsule expression, virulence, AdcAII, restriction modification, SpnD39III

## Abstract

The capsule is the dominant Streptococcus pneumoniae virulence factor, yet how variation in capsule thickness is regulated is poorly understood. Here, we describe an unexpected relationship between mutation of *adcAII*, which encodes a zinc uptake lipoprotein, and capsule thickness. Partial deletion of *adcAII* in three of five capsular serotypes frequently resulted in a mucoid phenotype that biochemical analysis and electron microscopy of the D39 *adcAII* mutants confirmed was caused by markedly increased capsule thickness. Compared to D39, the hyperencapsulated *ΔadcAII* mutant strain was more resistant to complement-mediated neutrophil killing and was hypervirulent in mouse models of invasive infection. Transcriptome analysis of D39 and the *ΔadcAII* mutant identified major differences in transcription of the Sp_0505-0508 locus, which encodes an SpnD39III (ST5556II) type I restriction-modification system and allelic variation of which correlates with capsule thickness. A PCR assay demonstrated close linkage of the SpnD39IIIC and F alleles with the hyperencapsulated *ΔadcAII* strains. However, transformation of *ΔadcAII* with fixed SpnD39III alleles associated with normal capsule thickness did not revert the hyperencapsulated phenotype. Half of hyperencapsulated *ΔadcAII* strains contained the same single nucleotide polymorphism in the capsule locus gene *cps2E*, which is required for the initiation of capsule synthesis. These results provide further evidence for the importance of the SpnD39III (ST5556II) type I restriction-modification system for modulating capsule thickness and identified an unexpected linkage between capsule thickness and mutation of *ΔadcAII*. Further investigation will be needed to characterize how mutation of *adcAII* affects SpnD39III (ST5556II) allele dominance and results in the hyperencapsulated phenotype.

## Abstract (precis)

The Streptococcus pneumoniae capsule affects multiple interactions with the host including contributing to colonization and immune evasion. During infection, the capsule thickness varies, but the mechanisms regulating this are poorly understood. We have identified an unsuspected relationship between mutation of *adcAII*, a gene that encodes a zinc uptake lipoprotein, and capsule thickness. Mutation of *adcAII* resulted in a striking hyperencapsulated phenotype, increased resistance to complement-mediated neutrophil killing, and increased S. pneumoniae virulence in mouse models of infection. Transcriptome and PCR analysis linked the hyperencapsulated phenotype of the *ΔadcAII* strain to specific alleles of the SpnD39III (ST5556II) type I restriction-modification system, a system which has previously been shown to affect capsule thickness. Our data provide further evidence for the importance of the SpnD39III (ST5556II) type I restriction-modification system for modulating capsule thickness and identify an unexpected link between capsule thickness and *ΔadcAII*, further investigation of which could further characterize mechanisms of capsule regulation.

## INTRODUCTION

Streptococcus pneumoniae (the pneumococcus) is a Gram-positive bacterial commensal of the human nasopharynx (1) and also a common invasive pathogen causing pneumonia, septicemia, and meningitis (2). S. pneumoniae has multiple virulence factors which facilitate disease pathogenesis (3), the most important of which is the capsule. The capsule is an extracellular polysaccharide layer which plays a crucial role in S. pneumoniae immune evasion by inhibiting complement recognition, phagocytosis, and bacterial entrapment by mucus (4). Variation in S. pneumoniae capsule structure results in multiple different biochemical and antigen structures, with at least 98 distinct capsule polysaccharide serotypes recognized at present (5). This diversity is mainly related to genetic variation in the multigene *cps* locus (6) and correlates closely with strain phenotypes such as invasive potential, duration of colonization, and ability to evade complement-mediated neutrophil phagocytosis (7, 8). The degree of capsule expression by S. pneumoniae is also affected by phase variation at different sites of infection (9, 10). Opaque-phase S. pneumoniae has increased thickness of the capsule layer and is associated with invasive infections such as septicemia, whereas transparent-phase S. pneumoniae has thinner capsule layers and is associated with colonization and biofilm formation (11–13). Despite the importance of capsule expression during S. pneumoniae interactions with the host, the molecular mechanisms underpinning phase variation and capsule thickness remain relatively poorly understood.

One mechanism that has been recently described to control capsule expression is epigenetic regulation by phase-variable control of DNA methylation driven by the type I restriction-modification system SpnD39III (ST5556II) (14). The SpnD39III (ST5556II) system consists of multiple genes that can be shuffled by recombination on inverted repeats to create enzymes capable of methylation at six different recognition sites. Capsule expression and thickness (opaque versus transparent) have been correlated with different SpnD39III alleles (14–16), and this system may be involved in regulating at least some aspects of S. pneumoniae phase variation. As yet, both the environmental conditions influencing allele distribution and how the effects of methylation patterns on gene expression lead to changes in capsule thickness have not been resolved.

Within mammalian hosts, the available concentrations of several cations are strictly controlled. As a consequence, cation ABC transporters of iron, manganese, and zinc are essential for S. pneumoniae growth and survival in the host (17–19). ABC transporters consist of a membrane-attached lipoprotein substrate binding protein and membrane permease(s) and ATPase proteins. Zinc acquisition is mediated by two ABC transporters identified by their lipoprotein components as AdcA and AdcAII (20, 21). Adjacent to *adcAII* is *phtD*, which encodes the surface protein PhtD, a member of the Pht histidine triad surface protein family that are involved in S. pneumoniae virulence. The histidine triad motifs of Pht proteins have a high affinity for zinc, and these proteins may provide a surface reservoir of zinc for import into S. pneumoniae via AdcA and AdcAII ABC transporters (22–24). We have previously demonstrated that deletion of *adcA* partially attenuates virulence, and deletion of both *adcA* and *adcAII* had a profound effect on S. pneumoniae physiology under low zinc conditions and strongly attenuated virulence (19, 25). In contrast, the virulence of the single *adcAII* deletion mutant was significantly increased. Here, we describe this unexpected consequence of partial deletion of *adcAII* in detail and show that the hypervirulence of the D39 *ΔadcAII* mutant strains is associated with a mucoid phenotype and increased capsule expression and is correlated closely with specific SpnD39III alleles and a point mutation in the *csp2E* capsule locus gene.

## RESULTS

### Deletion of *adcAII* in the S. pneumoniae D39 strain results in a markedly increased expression of the capsule.

**FIG 1:**
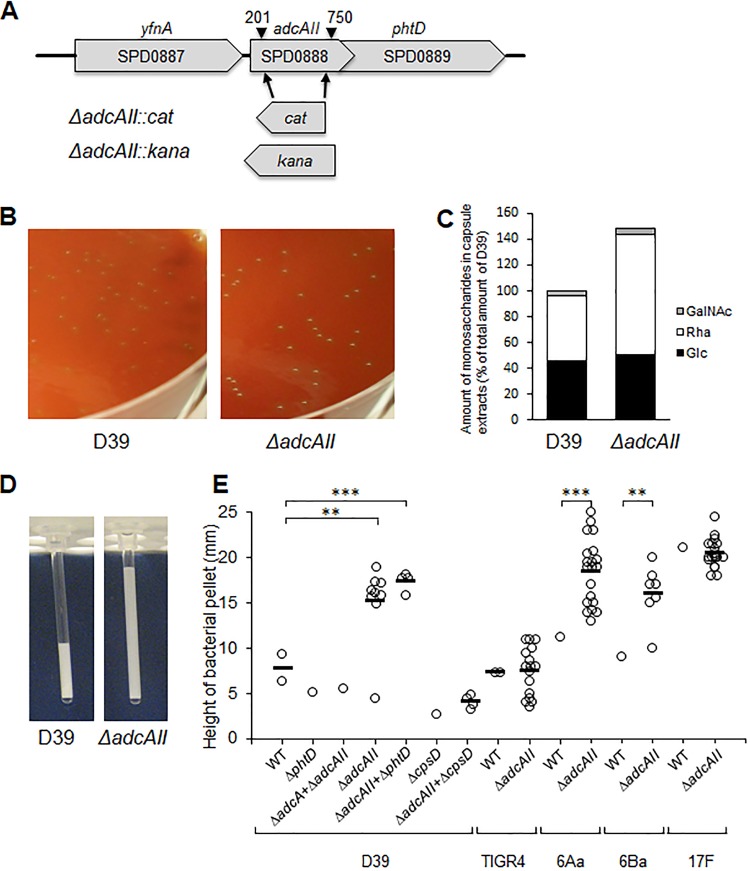
Creation and macroscopic phenotype of the Δ*adcAII* mutant. (A) Gene map of the *adcAII* locus showing the bp 201 to 750 deletion and replacement with an antibiotic resistance cassette (*cat* or *kana*) present in the Δ*adcAII* mutant. (B) Colony morphology on Columbia blood agar plates of wild-type (WT) D39 strain and the Δ*adcAII* mutant. (C) Relative amount of monosaccharides in capsule extracts of WT D39 strain and Δ*adcAII* mutant determined by GC-MS. All monosaccharide derivatives were identified according to their specific retention times and EI-MS fragmentations, as described in reference 26. (D) Example of measuring the volume of D39 and Δ*adcAII* bacterial pellets using microcapillary tubes. (E) Height (mm) of bacterial pellets for the WT and mutant strains in the indicated strains measured using microcapillary tubes. Each point represents data for independent clones containing the indicated mutation, and bars represent mean values for independently derived colonies for each mutant strain. *P* values were calculated using unpaired *t* test. **, *P* < 0.01; ***, *P* < 0.001.

**FIG 2:**
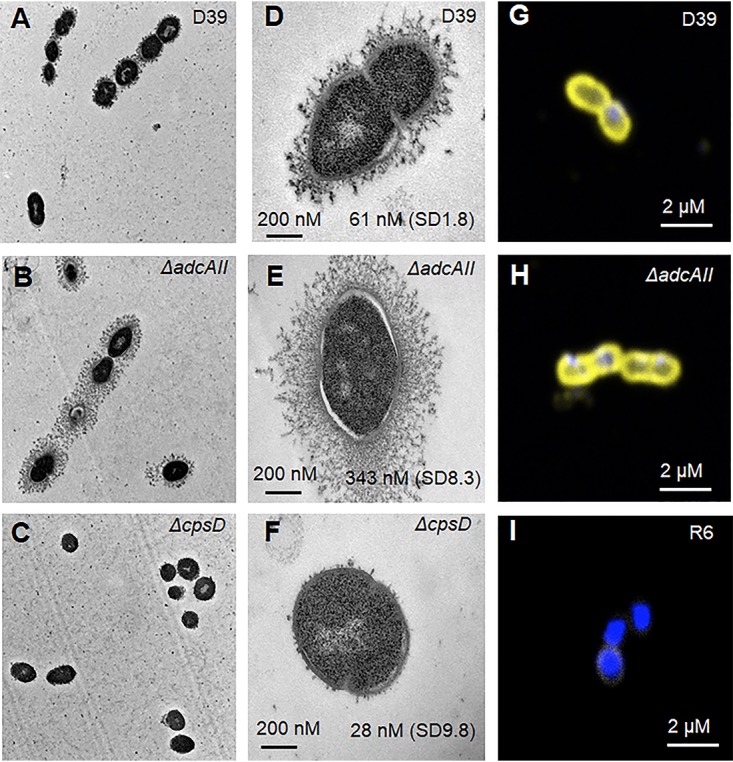
Microscopic phenotype of the Δ*adcAII* mutant. (A, D, and G) Wild type. (B, E, and H) Δ*adcAII* mutant. (C and F) Δ*cpsD* mutant. (I) R6 unencapsulated strain. (A to F) Electron microscopy of WT D39 strain and mutants, showing ultrathin sections of pneumococcus after capsule spring fixation using lysine-acetate-based ruthenium red-osmium protocol. Scale bars and mean capsule width in nm (SD) are given in the closeup views of selected examples of each strain in the right-hand column. (G to I) Confocal microscopy of wild-type D39, R6, and Δ*adcAII* mutant strains showing the capsule in yellow (anti-type 2 capsule antibody and Alexa Fluor 546 anti-rabbit antibody) and DAPI in blue.

During our previous investigation of the functional roles of the AdcA and AdcAII zinc ABC transporter systems, a single deletion mutant of the *adcAII* gene was made by partial replacement of the *adcAII* gene with the chloramphenicol resistance cassette *cat* (Fig. 1A). The resulting *ΔadcAII* mutant strains displayed a visibly increased mucoid colony morphology (Fig. 1B). Capsule thicknesses were compared between the D39 and *ΔadcAII* mutant strains using a range of assays. Initially, colony volume was assessed by transferring single colonies to a capillary tube and measuring the height of visible bacterial material. This demonstrated an increased volume of the *ΔadcAII* strain compatible with a thicker capsule layer (Fig. 1D and E). Capsule width was then directly visualized for the *ΔadcAII* and wild-type D39 strains using electron microscopy (EM), which demonstrated that the bacterial cells of the *ΔadcAII* mutant had a considerably enlarged capsule layer compared to D39 (Fig. 2A to F). The mean capsule radius indicated that the Δ*adcAII* mutant expressed a capsule 5.6 times thicker than the wild-type (WT) D39 (capsule width of 61 ± 1.8 nm versus 343 ± 8.3 nm for the D39 and Δ*adcAII* strains, respectively; *n* = 30 for each strain). Monosaccharide composition of capsule extracts for the Δ*adcAII* mutant and WT D39 strain extracts were assessed biochemically using gas chromatography-mass spectrometry (GC-MS). Total polysaccharide in capsule extracts demonstrated a 1.5-fold increase in the Δ*adcAII* mutant compared to the wild-type strain, largely due to a 2-fold increase in rhamnose content (Fig. 1C). Despite these changes in polysaccharide content, the hyperencapsulated Δ*adcAII* strain was still recognized by serotype-specific antisera (Fig. 2G to I). The small amount of GalNac detected was probably from teichoic acids extracted with the capsular polysaccharide. Overall, these data demonstrated that partial deletion of *adcAII* modified the polysaccharide content of the capsule with overexpression of rhamnose-containing polysaccharides. To assess whether the Δ*adcAII* mutant phenotype was serotype specific, additional Δ*adcAII* mutant strains were obtained in capsular serotype 4, 6A, 6B, and 17F strains. Partial deletion of *adcAII* in the 6A and 6B serotypes also resulted in a mucoid phenotype suggestive of increased capsule thickness but did not affect capsule thickness in the serotype 4 and 17F strains (Fig. 1E).

### Consistent association of the *adcAII* mutation with increased capsule expression by D39.

To characterize further the relationship between partial deletion of *adcAII* and increased capsule thickness, additional transformation and phenotyping experiments were performed. Increased capsule expression was also detected in *ΔadcAII* strains made using the kanamycin resistance cassette *kana* instead of *cat* and if the deletion included the immediate downstream gene (*phtD*) (Fig. 1E and Table 1). Combined deletion of *adcA* and *adcAII* did not result in an increased capsule thickness phenotype. When the *adcAII* mutation was created in an unencapsulated D39 strain (Δ*cpsD*), colony volumes were similar to the parental strain and markedly lower than with *ΔadcAII* mutations in the WT D39 strain (Fig. 1E). The frequency with which deletion of *adcAII* resulted in a strain with an increased capsule thickness was investigated using multiple transformants made using the *adcAII* deletion constructs or by transformation with genomic DNA extracted from a *ΔadcAII* strain mutant. Of the 100 transformants, 44% (*kana*) or 42% (*cat*) had increased capsule thickness when transformed with the PCR construct and 78% (18 out of 23) when transformed with genomic DNA (Table 1). The remaining mutant clones either had a normal capsule thickness or were unencapsulated. Growth of the *ΔadcAII* strain in chemically defined medium (CDM) supplemented with 33 μM cations (Mn^2+^ or Zn^2+^), 5% sucrose, or recombinant PhtD (50 μg/ml) or in CDM depleted of cations by treatment with 1 mM EDTA did not reduce increased capsule expression (data not shown; measured using capillary tube colony volume). The increased capsule thickness phenotype was stable, with 100% of 100 colonies retaining a thick capsule after a single mucoid colony was cultured in THY (Todd-Hewitt broth supplemented with yeast extract) liquid medium followed by plating on blood agar plates over five generations. These data show that transformation of the S. pneumoniae D39 strain with a deletion construct affecting *adcAII* frequently results in transformants with a marked increase in capsule quantity.

**TABLE 1:** Δ*adcAII* mutant method of construction/source of DNA for the targeted deletion related to the capsule phenotype for multiple transformants

DNA source for transformation	No. of clones analyzed	Capsule phenotype		
		Absent^*a*^	Normal	Thick
PCR fragment *adcAII*::*kana*	100	14 (1)	32	44
PCR fragment *adcAII*::*cat*	100	45 (4)	13	42
Genomic DNA R6 Δ*adcAII*::*cat1* 1st	4	0	0	4
Genomic DNA R6 Δ*adcAII*::*cat1* 2nd	4	1	0	3
Genomic DNA R6 Δ*adcAII*::*cat2*	15	1	3	11

a Numbers in parentheses are numbers of absent capsule strains sequenced all of which contained the Q308 stop codon mutation in *cps2E*.

### The hyperencapsulated D39 Δ*adcAII* strain is resistant to complement-mediated phagocytosis.

**FIG 3:**
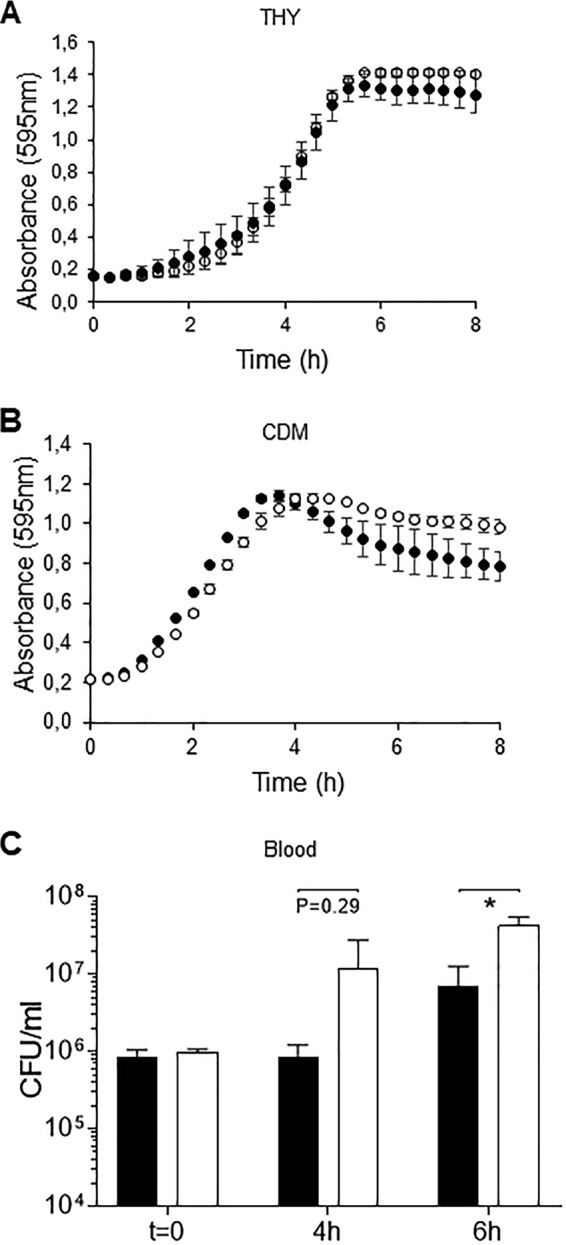
Growth phenotype of the WT D39 strain and Δ*adcAII* mutant. (A and B) Bacteria were inoculated at an OD_595_ of 0.01 in THY (A) or CDM (B) supplemented with 33 μM Zn and incubated at 37°C for 8 h. Black circles, WT D39; white circles, Δ*adcAII* mutant. Two independent assays were performed using triplicate wells. Each point is the mean (SD) for the results of a representative experiment. (C) Mean (SD) WT D39 or Δ*adcAII* mutant CFU after culture in blood (1 ml inoculated with 1 × 10^6^ CFU) for 4 and 6 h. *P* values were calculated using unpaired Student’s *t* test. *, *P* < 0.05.

**FIG 4:**
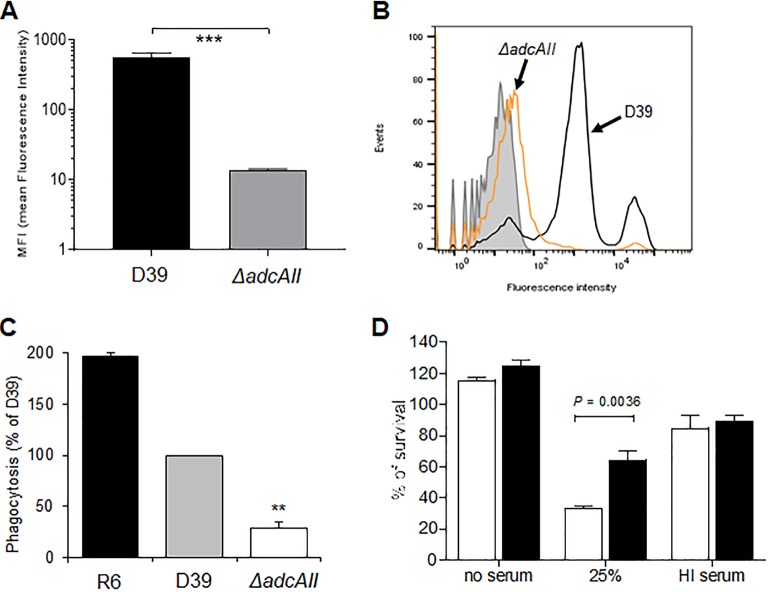
The Δ*adcAII* mutant has increased resistance to complement and phagocytosis. (A) Mean fluorescence index (MFI; measured in arbitrary units) of C3b/iC3b deposition on WT D39 or Δ*adcAII* mutant measured using flow cytometry in 25% human serum. Error bars represent SDs. ***, *P* < 0.001, unpaired *t* test. (B) Examples of flow cytometry histograms for C3b/iC3b deposition on WT D39 or Δ*adcAII* mutant in 100% human serum. Gray shadowing indicates the results for bacteria incubated in PBS alone. (C) Flow cytometry quantification of macrophage (THP-1 cells) phagocytosis of isothiocyanate fluorescein-labeled WT D39, R6 (unencapsulated derivative of D39), and the Δ*adcAII* mutant for 1 h at 37°C (50 CFU/cell). The percentage of fluorescent macrophages was quantified by flow cytometry, and the data are expressed as means (SD) of the percentage of the results for the WT D39 strain. **, *P* < 0.01, unpaired Student’s *t* tests. (D) Mean proportions of WT D39 (white columns) and the Δ*adcAII* mutant (black columns) surviving incubation with fresh human neutrophils for 45 min (MOI of 500 bacteria/neutrophil). Data are given for bacteria preincubated in PBS, 25% normal human serum, or 25% heat-inactivated human serum (no complement activity). Error bars represent SDs, and *P* values were obtained using unpaired *t* tests.

**FIG 5:**
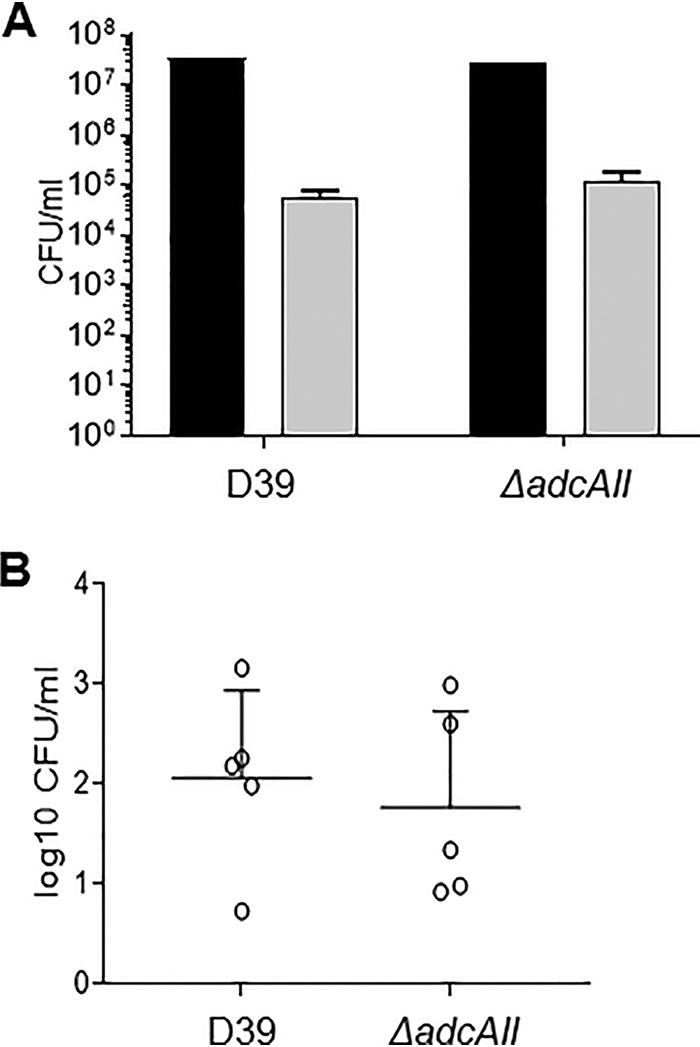
Effects of Δ*adcAII* on adhesion to epithelial cells. (A) Bacterial CFU recovered from the Detroit 562 cell adhesion assay (duration 1 h) with WT D39 or Δ*adcAII* mutant. There were no statistical differences in CFU recovered for each strain (unpaired *t* tests). (B) WT D39 or Δ*adcAII* mutant CFU in nasal washes recovered from mice 5 days after inoculation of either strain with 10^7^ CFU under light halothane general anesthesia. Each symbol represents data from a single mouse, bars represent medians, and error bars represent the upper interquartile range. There were no statistically significant differences in nasal wash CFU.

The capsule is an essential virulence factor that prevents opsonophagocytosis of S. pneumoniae but at a metabolic cost during S. pneumoniae growth (7, 26). We therefore investigated the phenotypes of the hyperencapsulated D39 *ΔadcAII* strain *in vitro* and in murine infection models. Growth of the *ΔadcAII* strain was similar to the WT D39 in complete medium THY and in CDM (supplemented with 33 μM zinc to overcome effects of loss of *adcAII* on zinc transport) (Fig. 3A and B). In contrast, in blood approximately 1 log_10_ more *ΔadcAII* bacteria were recovered after 4 h of incubation compared to the D39 WT strain, with large differences in CFU persisting at 6 h (Fig. 3C). Flow cytometry demonstrated increased resistance to opsonization with complement and macrophage phagocytosis of the D39 *ΔadcAII* strain compared to the D39 WT strain (Fig. 4A to C). The D39 *ΔadcAII* strain also had increased resistance to killing by neutrophils compared to the WT strain; these differences were lost if bacteria were opsonized in heat-treated (i.e., complement-deficient) sera or in phosphate-buffered saline (PBS) alone, demonstrating that the differences were largely complement dependent (Fig. 4D). Adhesion assays showed there was no defect for the D39 *ΔadcAII* strain in binding to the respiratory epithelium cell line Detroit 562 compared to the WT strain (Fig. 5A). Hence, increased capsule expression by the *ΔadcAII* strain was associated with resistance to complement-mediated phagocytosis but did not inhibit adhesion to a human nasopharyngeal cell line.

### The hyperencapsulated Δ*adcAII* strain has increased virulence.

**FIG 6:**
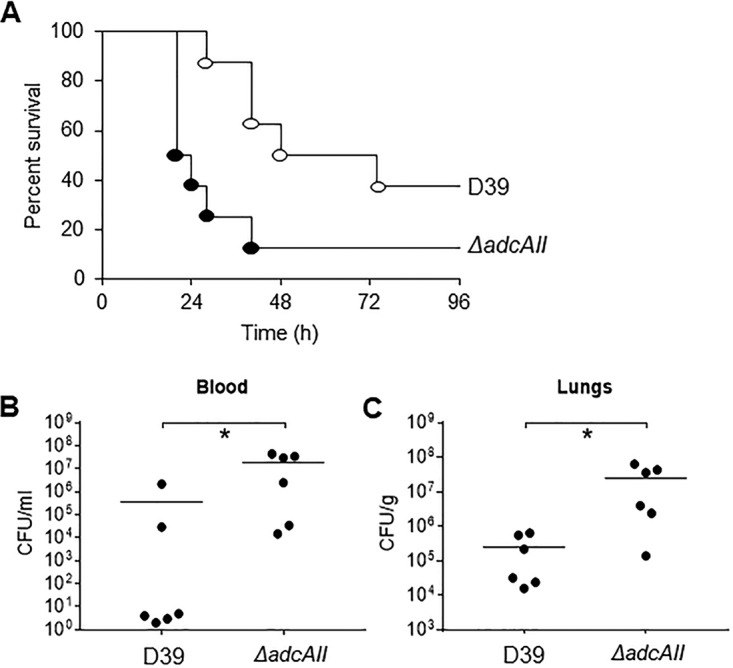
The Δ*adcAII* mutant has increased virulence in mouse models of sepsis and pneumonia. (A) For the septicemia model, 5 × 10^4^ CFU of each strain was injected intraperitoneally and the progress of infection was followed over time. Empty circles represent data for the WT D39 strain, and filled black circles show the Δ*adcAII* mutant strain. *P* values were obtained using the log rank test. (B and C) Blood (B) and lung (C) CFU for the pneumonia model determined by plating serial dilutions on Columbia blood agar recovered 48 h after infection for mice inoculated by intranasal instillation of 5 × 10^6^ CFU. Each symbol represents data from a single mouse, bars represent medians, and *P* values were calculated using unpaired *t* tests. *, *P* < 0.05.

Both colony forming units (CFU) in nasal washes at day 5 and competitive infection experiments demonstrated that the hyperencapsulated D39 *ΔadcAII* strain colonized the nasopharynx to a similar degree as the WT D39 (Fig. 5B; Table 2), results which are consistent with the lack of a difference between the strains for adhesion to Detroit 562 cells. In contrast, the hyperencapsulated *ΔadcAII* strain had increased virulence during systemic or pneumonic infection. In competitive infection experiments using a sepsis model (intraperitoneal [i.p.] inoculation), the D39 *adcAII* strain strongly outcompeted the WT strain (Table 2), and in a murine sepsis model using pure inocula of each strain, 80% of mice infected with the D39 *ΔadcAII* strain progressed to fatal infection by 40 h compared to 40% of mice infected with WT D39 (Fig. 6A). Finally, in a pneumonia model higher CFU was recovered in both the lungs and blood from mice infected with the *ΔadcAII* mutant compared to wild-type D39 (Fig. 6B and C).

**TABLE 2:** Competitive index data for infection models using a mixed inoculum of 50% WT D39 and 50% D39 *ΔadcAII* hyperencapsulated strain

Infection model	Inoculation route and CFU	Sample source (time point)	CI (SD)	*n*	*P* value
Nasopharyngeal colonization	Intranasal, 5 × 10^6^ CFU	Nasal washes (5 days)	1.04 (0.15)	4	0.58
Sepsis	Intraperitoneal, 5 × 10^4^ CFU	Blood (24 h)	4.6 (0.62)	7	<0.0001

### Transcriptome analysis of wild-type and hyperencapsulated Δ*adcAII* strains.

To investigate mechanisms causing increased capsule production by the *ΔadcAII* strain, a transcriptome microarray analysis was performed on WT D39, one hyperencapsulated *ΔadcAII* and one *ΔadcAII/phtD* strain clone, and one *ΔadcAII*::*cat* unencapsulated clone (Cl44) (Table 3). Three independent RNA extracts for each strain were submitted to transcriptomic analysis. In total, 89 genes showed significant changes in expression (>1.5-fold, *P* < 0.05) between the wild-type D39 and hyperencapsulated *ΔadcAII* strain (78 with reduced and 11 with increased expression in the mutant strain including the deleted *adcAII* and downstream *phtD* genes), 96% (86/89) of which also showed comparable changes in expression in the thick-capsule *ΔadcAII/phtD* strain. In contrast, 11% (10/89) of these genes showed similar changes in expression in the Cl44 *ΔadcAII* strain without increased capsule expression, suggesting that the gene expression changes were linked to the capsule phenotype. Expression of the D39 capsule locus genes was not significantly different between the strains. Genes showing increased expression in the hyperencapsulated *ΔadcAII* and *ΔadcAII/phtD* strains included genes related to zinc uptake (*adcR*, *adcA*, *phtA*, and *phtE*), suggesting compensatory effects due to loss of the AdcAII zinc transporter. The other genes showing increased expression in the hyperencapsulated strains encode proteins of unknown function or containing LysM domains predicted to be involved in cell wall metabolism (27). Three of the operons that showed reduced expression in the hyperencapsulated strains are predicted to be involved in pyrimidine synthesis: SPD_0608-09, encoding a predicted orotate decarboxylase and phosphoribosyltransferase and being part of a larger operon encompassing SPD_0608 to SPD_06187 (28); SPD_0851-52, predicted to encode a dihydroorotate dehydrogenase electron transfer subunit (29); and SPD_1131, predicted to encode a carbamoylphosphate synthase large subunit required for pyrimidine synthesis from glutamine (30). Other genes showing reduced expression in the hyperencapsulated strains have roles in iron uptake (SPD_0224, -0226, and -1650), carbohydrate uptake (SPD_0279, 0362, 1050-1053, 1501, and 1987-95), and riboflavin synthesis (SPD_0166-69). Of particular interest, the hyperencapsulated strains showed reduced expression of SPD_0450, SPD_0452, and SPD_0453, *creX* (*psrA*), *hdsS*′ (*hsdS2*), and *hdsS* (*hsdS1*), from the SpnD39III (ST5556II) type I restriction-modification system, *respectively;* this is discussed in detail below.

**TABLE 3:** Relative gene expression detected by microarray for genes showing statistically significant >1.5-fold differences in expression for the thick-capsule *ΔadcAII* AIIL strain compared to the WT D39 strain^*a*^

Regulation status in hyperencapsulated strains and gene no.	Gene name	Predicted/known function	Mutant strain and capsule phenotype		
			Δ*adcAII* (AIIL) thick	Δ*adcAII*/*phtD* (AII+Pcl4) thick	Δ*adcAII* (Cl44) none
Upregulated					
SPD_0104		LysM domain protein	1.80	1.93	1.20
SPD_0389	*accD*	Acetyl-CoA carboxylase subunit beta	1.55	1.59	−*1.37*
SPD_0646		Hypothetical protein	1.74	2.05	−*1.18*
SPD_0890	*phtE*	Histidine triad protein	3.04	3.18	*1.04*
SPD_0891		Truncated histidine triad protein	3.25	*1.16*	*1.37*
SPD_0892		Truncated histidine triad protein	3.31	*1.09*	*1.43*
SPD_0893		Hypothetical protein	3.39	*1.04*	*1.59*
SPD_1038	*phtA*	Histidine triad protein	1.98	1.52	−*1.05*
SPD_1874		LysM domain-containing protein	2.53	2.90	*1.19*
SPD_1997	*adcA*	Zinc ABC transporter AdcA lipoprotein	1.52	1.41	1.03
SPD_2000	*adcR*	*adc* operon repressor AdcR	1.55	1.65	−*1.06*

Downregulated					
SPD_0052	*purL*	Phosphoribosylformylglycinamidine synthase	−2.45	−2.36	*1.24*
SPD_0053	*purF*	Amidophosphoribosyltransferase	−2.27	−2.34	*1.25*
SPD_0055	*purN*	Phosphoribosylglycinamide formyltransferase	−2.13	−2.18	*1.36*
SPD_0090		ABC transporter lipoprotein	−2.02	−2.19	*2.04*
SPD_0166	*ribH*	Riboflavin synthase, beta subunit	−2.76	−3.44	*1.56*
SPD_0167	*ribB*	Riboflavin biosynthesis protein RibB	−2.51	−3.34	*1.71*
SPD_0168	*ribE*	Riboflavin synthase subunit alpha	−2.52	−3.30	*1.66*
SPD_0169	*ribD*	Riboflavin biosynthesis protein RibD	−2.40	−3.30	*1.67*
SPD_0224	*pitD*	PitD iron ABC transporter permease	−2.28	−2.11	*1.46*
SPD_0226	*pitA*	PitA iron ABC transporter lipoprotein	−2.01	−1.37	*1.72*
SPD_0265	*adhP*	Alcohol dehydrogenase	−1.80	−1.83	*1.63*
SPD_0279	*celB*	Cellobiose PTS system IIB component	−2.28	−1.79	*1.76*
SPD_0300		Oligohyaluronate lyase	−2.49	−1.60	*1.32*
SPD_0362	*mtlF*	Mannitol PTS system IIA component	−2.38	−2.40	*1.40*
SPD_0364		Amino acid ABC transporter ATPase	−3.00	−2.80	*1.84*
SPD_0444	*lytB*	Endo-beta-*N*-acetylglucosaminidase	−1.55	−1.69	*1.38*
**SPD_0450**	***creX/psrA***	**Type I restriction-modification system**	**−3.39**	**−4.24**	***−1.43***
**SPD_0452**	***hsdS*′ (*hsdS2*)**	**Type I restriction-modification system**	**−3.62**	**−6.41**	***1.25***
**SPD_0453**	***hsdS* (*hsdS1*)**	**Type I restriction-modification system**	**−2.01**	**−2.36**	***1.15***
SPD_0466	*blpT*	BlpT protein, fusion	−1.79	−2.26	*1.52*
SPD_0472	*blpA*	ABC transporter, ATP-binding protein	−2.21	−3.47	*1.59*
SPD_0473	*blpY*	Immunity protein BlpY	−1.50	−2.17	*1.44*
SPD_0553		Hypothetical protein	−1.59	−1.44	*1.26*
SPD_0595		Hypothetical protein	−1.55	−1.72	−0.64
SPD_0608	*pyrF*	Orotidine 5′-phosphate decarboxylase	−1.65	−1.60	*1.02*
SPD_0609	*pyrE*	Orotate phosphoribosyltransferase	−1.77	−1.67	*1.02*
SPD_0610		Hypothetical protein	−2.18	−2.26	*1.45*
SPD_0611		Hypothetical protein	−1.76	−1.94	*1.19*
SPD_0612		Hypothetical protein	−2.07	−2.06	*1.00*
SPD_0613		Hypothetical protein	−1.70	−1.83	*1.09*
SPD_0614		ABC transporter, ATP-binding protein	−1.76	−1.77	*1.11*
SPD_0615		ABC transporter substrate binding protein	−1.51	−2.25	*1.25*
SPD_0616	*glnQ*	Amino acid ABC transporter ATPase	−1.56	−2.38	*1.13*
SPD_0617	*glnP*	Amino acid ABC transporter permease	−1.76	−2.64	*1.25*
SPD_0618	*glnP*	Amino acid ABC transporter permease	−1.71	−2.51	*1.20*
SPD_0851	*pyrK*	Dihydroorotate dehydrogenase II	−1.90	−1.90	*1.16*
SPD_0852	*pyrD*	Dihydroorotate dehydrogenase IB	−2.32	−2.28	*1.11*
SPD_0853	*lytB*	Endo-beta-*N*-acetylglucosaminidase	−1.71	−1.65	*1.09*
**SPD_0888**	***adcAII***	**Zn^2+^ ABC transporter lipoprotein**	**−3.87**	**−3.03**	**−5.95**
**SPD_0889**	***phtD***	**Hypothetical protein**	**−1.78**	**−2.50**	**−*4.2***
SPD_1009	*serB*	Phosphoserine phosphatase	−1.51	−1.24	*1.60*
SPD_1011	*glxK*	Glycerate kinase	−1.63	−1.26	*1.58*
SPD_1035		PTS system, IIA component	−4.70	−4.83	−*1.91*
SPD_1036		PTS system, IIA component	−7.26	−5.84	−*2.93*
SPD_1050	*lacD*	Tagatose 1,6-diphosphate aldolase	−1.61	−1.60	*1.44*
SPD_1051	*lacC*	Tagatose-6-phosphate kinase	−1.62	−1.62	*1.49*
SPD_1052	*lacB*	Galactose-6-phosphate isomerase LacB	−1.58	−1.56	*1.46*
SPD_1053	*lacA*	Galactose-6-phosphate isomerase LacA	−1.61	−1.61	*1.51*
SPD_1074	*metY*	*O*-Acetylhomoserine sulfhydrylase	−1.64	−1.92	*1.51*
SPD_1131	*carB*	Carbamoylphosphate synthase subunit	−1.60	−1.37	*1.06*
SPD_1133	*pyrB*	Aspartate carbamoyltransferase subunit	−1.51	−1.30	*1.01*
SPD_1175		Putative membrane protein	−1.68	−1.75	−1.43
SPD_1176		ABC transporter, ATP-binding protein	−1.69	−1.81	*1.38*
SPD_1177		Drug efflux ABC transporter	−1.73	−1.68	*1.43*
SPD_1178	*ptrB*	Prolyl oligopeptidase family protein	−1.73	−1.78	−1.47
SPD_1179		Hypothetical protein	−1.74	−1.80	*1.59*
SPD_1454		Hypothetical protein	−1.56	−1.61	*1.17*
SPD_1455		Hypothetical protein	−1.84	−3.29	*1.25*
SPD_1498		Oxidoreductase	−2.21	−2.23	*1.34*
SPD_1501		Sugar ABC transporter permease	−3.73	−3.58	*1.42*
SPD_1503		Hypothetical protein	−3.25	−4.03	*1.09*
SPD_1513		Hypothetical protein	−1.77	−2.68	−1.40
SPD_1568		GTP cyclohydrolase	−1.80	−1.55	*1.53*
SPD_1584		ABC transporter permease	−2.30	−2.16	*2.08*
SPD_1650	*fatC*	Iron uptake ABC transporter permease	−2.84	−2.23	*1.30*
SPD_1793		Universal stress protein family	−1.54	−1.74	*2.00*
SPD_1865	*adh*	Zinc-containing alcohol dehydrogenase	−1.59	−1.28	*1.48*
SPD_1972		Hypothetical protein	−2.38	−2.96	*1.75*
SPD_1985	*adh2*	Iron-containing alcohol dehydrogenase	−2.05	−1.89	*1.59*
SPD_1987		Fucolectin-related protein	−3.12	−2.96	*1.49*
SPD_1989		PTS system, IID component	−2.19	−1.83	*1.88*
SPD_1990		PTS system, IIC component	−1.94	−1.74	*1.60*
SPD_1991		PTS system, IIB component	−1.83	−1.45	*1.52*
SPD_1992		PTS system, IIA component	−2.03	−1.79	*1.68*
SPD_1993	*fucU*	Fucose operon FucU protein	−2.33	−2.10	*1.83*
SPD_1994	*fucA*	l-Fuculose phosphate aldolase	−2.17	−2.33	*1.63*
SPD_1995	*fcsK*	l-Fuculose kinase FucK, putative	−2.13	−2.07	*2.12*
SPD_2013	*glpK*	Glycerol kinase	−2.76	−2.36	*1.25*

a For comparison, the fold differences compared to WT D39 strain for the thick-capsule Δ*adcAII*/*phtD* strain (AII + Pcl14) and a normal-capsule-thickness Δ*adcAII* strain (Cl144) are provided alongside. The *adcAII*, *phtD*, and SpnD39III (ST5556II) type I restriction-modification system genes are indicated in bold, and gene expression profile differences in the Δ*adcAII*/*phtD* or the Δ*adcAII* (Cl144) compared to the Δ*adcAII* AIIL hyperencapsulated strain are indicated in italics. Abbreviations: CoA, coenzyme A; PTS, phosphotransferase.

### Increased capsule thickness of the Δ*adcAII* strains correlated closely with specific *hsd* alleles.

**FIG 7:**
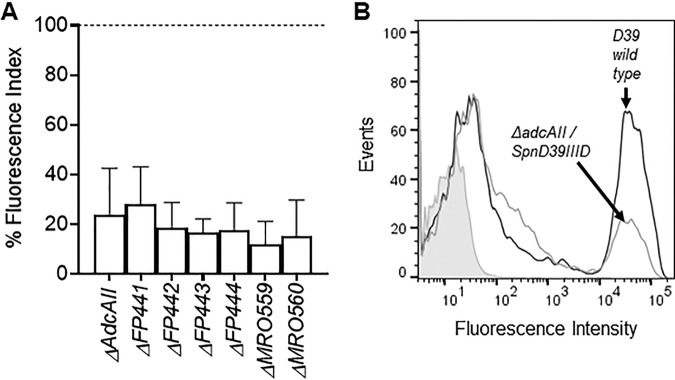
Flow cytometry analysis of complement sensitivity of the hyperencapsulated Δ*adcAII* strain after transformation with locked SpnD39III (ST5556II) alleles (A to F) containing an inactivated *creX* gene. Δ*FP441*, Δ*FP442*, Δ*FP443*, Δ*FP444*, Δ*MRO559*, and Δ*MRO560* are all double mutant strains carrying the *adcAII* mutation and an extra one in allele SpnIIIB, allele SpnIIIC, allele SpnIIIA, allele SpnIIID, allele SpnIIIE, and allele SpnIIIF, respectively. (A) Fluorescence index (MFI measured in arbitrary units multiplied by proportion of bacteria positive for C3b/iC3b) of C3b/iC3b deposition on Δ*adcAII* mutants and Δ*adcAII* fixed SpnD39III allele transformants (alleles A to F) as a proportion of the fluorescence index for the wild-type normal-capsule-thickness D39 strain. The data were measured using flow cytometry after preincubation in 30% human serum. Error bars represent SDs, and 10 transformants were tested for each double mutant strain. For all mutant strains, the *P* value for results compared to D39 was <0.001 (unpaired *t* tests). (B) Examples of flow cytometry histograms for C3b/iC3b deposition on WT D39 (dark gray line) and one Δ*adcAII/SpnD39IIID* allele (light gray line) double mutant transformant. Gray shading indicates the results for bacteria incubated in PBS alone.

The S. pneumoniae SpnD39III (ST5556II) type I restriction-modification locus undergoes genetic variation due to recombination within the locus between pairs of inverted repeats, generating six allelic variants which are linked to opaque (increased capsule expression) and transparent (reduced capsule expression) colony morphology (14–16). This suggests that the detected changes in expression of genes within the SpnD39III (ST5556II) locus could reflect differences in the proportions of the allelic variants between the WT and *ΔadcAII* strains, and these differences could underpin the hyperencapsulated phenotype of the latter. Hence, the proportion of each of the six SpnD39III (ST5556II) variants was obtained for multiple individual *ΔadcAII* strains expressing either thick or normal-size capsules using a previously described assay based on PCR followed by restriction digestion of the products (14) (Table 4). This showed a clear correlation between capsule phenotype and the dominant SpnD39III (ST5556II) variant. The WT D39 strain contained a mixture of the SpnD39III (ST5556II) variants, mainly SpnD39IIIC with also a significant proportion of the SpnD39IIID and F variants. With one exception, SpnD39IIIC (3 strains) and F (5 strains) were the dominant variants found in the hyperencapsulated *ΔadcAII* strains, whereas SpnD39IIID (7 strains) or A (1 strain) was the dominant variant found in the *ΔadcAII* strains with normal capsule thickness. To try to link increased capsule formation by some *ΔadcAII* mutants to changes in the dominant alleles of the SpnD39III (ST5556II) type I restriction-modification locus, the hyperencapsulated *ΔadcAII* strain was transformed with genomic DNA from D39 mutant strains with locked SpnD39III (ST5556II) alleles due to an inactivated *creX* gene. Flow cytometry analysis of complement sensitivity was used to rapidly assess capsular phenotype for 10 transformants for each allele (A to F). All transformants retained the complement-resistant phenotype of the hyperencapsulated *ΔadcAII* strain, even those made using the SpnD39III (ST5556II) alleles associated with a normal capsule width in *ΔadcAII* transformants (A and D) (Fig. 7), suggesting they all remained hyperencapsulated.

**TABLE 4:** Proportions of variants (identified by PCR analysis) for the SpnD39III (ST5556II) type I restriction-modification system for selected *ΔadcAII* mutant strains divided into those with thick and normal capsule thicknesses

Phenotype	Strain	Proportion (%) of SpnD39III (ST5556II) variant:					
		A	B	C	D	E	F
Wild type	D39	2.2	0	67.2	15.6	0	15.0

Thick capsule	Cl82	1.3	0	3.8	7.1	1.0	86.8
	Cl72	1.7	0	4.3	9.2	0	84.8
	Cl10	1.3	0	4.0	8.8	0	85.9
	Cl38	2.0	0	1.9	8.4	2.3	85.4
	Cl3 2P	1.0	0	2.5	6.4	1.6	88.6
	Cl5 1P	0.7	0	87.8	9.8	0	1.7
	Cl3 1P	1.7	0	84.8	10.3	0	3.3
	Cl1 1G	2.2	0	83.1	11.1	0	3.7
	Cl7 1G	0	0	7.7	92.3	0	0
	AIIL	3.8	0.6	74.9	17.8	0.0	2.8

Normal capsule	Cl88	0	0	6.0	92.7	0.00	1.3
	Cl28	0	0	6.7	93.3	0	0
	Cl35	0	0	6.92	93.1	0	0
	Cl6	0	0.7	5.71	93.6	0	0
	Cl73	0	0	9.88	90.1	0	0
	Cl20	0	0	9.93	90.1	0	0
	Cl17	0	9.0	2.9	88.2	0	0
	Cl1	0.4	1.7	16.4	80.8	0	0.6
	Cl36	94.3	5.8	0	0	0	0

### Genome sequence data for Δ*adcAII* strains.

Genome sequencing of one *ΔadcAII* and one *ΔadcAII*/*pht* strain confirmed they contained the expected partial deletion of *adcAII* or *adcAII* and *phtD*, respectively, with insertion of the antibiotic resistance cassette with no additional mutations elsewhere in the genome. Sequencing of the *cps* locus in additional *ΔadcAII* strains found that a nonsynonymous single nucleotide polymorphism (SNP) affecting the capsule locus gene *cps2E* (E to K at amino acid 322) was present in five out of 10 clones (Table 5), suggesting that this SNP may be relevant for the hyperencapsulated phenotype at least for a proportion of *ΔadcAII* strains. However, the same SNP was also present in one of eight *ΔadcAII* strains with normal capsule thickness. All five of the unencapsulated *ΔadcAII* strains investigated had *cps2E* genes containing a stop codon at amino acid 308, which could explain their unencapsulated phenotype (Table 5).

**TABLE 5:** Mutation construction, capsule phenotype, and (where available) *cps2E* gene genome sequence data for S. pneumoniae strains

Strain/clone	Gene deletion	Antibiotic resistance	Mutant construction	Capsule ratio/D39	Capsule phenotype	Mutation in *cps2E*
D39 800	None			1	Normal	None
D39 WT	None			1	Normal	None
*ΔadcAII*L	*ΔadcAII*	Cm	New transformation	3.7	Thick	None
Cl10	*ΔadcAII*	Kana	New transformation	3	Thick	E for K aa^*a*^ 322
Cl57	*ΔadcAII*	Kana	New transformation	0.5	Unencapsulated	Stop codon aa 308
Cl1 1P	*ΔadcAII*	Cm	Back-crossing with *ΔadcAII*L	0.5	Unencapsulated	Not sequenced
Cl1 1G	*ΔadcAII*	Cm	Back-crossing with *ΔadcAII*L	3	Thick	Not sequenced
Cl1 1G	*ΔadcAII*	Cm	Back-crossing with *ΔadcAII*L	2.9	Thick	None
Cl2 1P	*ΔadcAII*	Cm	Back-crossing with *ΔadcAII*L	0.5	Unencapsulated	Not sequenced
Cl2 2P	*ΔadcAII*	Cm	Back-crossing with *ΔadcAII*L	0.9	Normal	Not sequenced
Cl3 1G	*ΔadcAII*	Cm	Back-crossing with *ΔadcAII*L	3.1	Thick	Not sequenced
Cl3 1P	*ΔadcAII*	Cm	Back-crossing with *ΔadcAII*L	3.2	Thick	Not sequenced
Cl3 2P	*ΔadcAII*	Cm	Back-crossing with *ΔadcAII*L	3.1	Thick	None
Cl5 1P	*ΔadcAII*	Cm	Back-crossing with *ΔadcAII*L	3.8	Thick	None
Cl5 1G	*ΔadcAII*	Cm	Back-crossing with *ΔadcAII*L	3.7	Thick	Not sequenced
Cl6 1P	*ΔadcAII*	Cm	Back-crossing with *ΔadcAII*L	0.5	Unencapsulated	Not sequenced
Cl6 2P	*ΔadcAII*	Cm	Back-crossing with *ΔadcAII*L	0.5	Unencapsulated	Stop codon aa 308
Cl7 1P	*ΔadcAII*	Cm	Back-crossing with *ΔadcAII*L	3.5	Thick	Not sequenced
Cl7 1G	*ΔadcAII*	Cm	Back-crossing with *ΔadcAII*L	3.1	Thick	None
AIIcl 1	*ΔadcAII*	Cm	New transformation	1	Normal	None
AIIcl 17	*ΔadcAII*	Cm	New transformation	1.1	Normal	None
AIIcl 20	*ΔadcAII*	Cm	New transformation	1.1	Normal	None
AIIcl 28	*ΔadcAII*	Cm	New transformation	1.15	Normal	None
AIIcl 31	*ΔadcAII*	Cm	New transformation	0.5	Unencapsulated	Stop codon aa 308
AIIcl 35	*ΔadcAII*	Cm	New transformation	0.85	Unencapsulated	Stop codon aa 308
AIIcl 36	*ΔadcAII*	Cm	New transformation	1.05	Normal	E for K aa 322
AIIcl 38	*ΔadcAII*	Cm	New transformation	2.05	Thick	E for K aa 322
AIIcl 44	*ΔadcAII*	Cm	New transformation	0.5	Unencapsulated	Stop codon aa 308
AIIcl 72	*ΔadcAII*	Cm	New transformation	2.8	Thick	E for K aa 322
AIIcl 73	*ΔadcAII*	Cm	New transformation	1.15	Normal	None
AIIcl 75	*ΔadcAII*	Cm	New transformation	1.2	Normal	None
AIIcl 78	*ΔadcAII*	Cm	New transformation	1.8	Thick	E for K aa 322
AIIcl 82	*ΔadcAII*	Cm	New transformation	2.6	Thick	E for K aa 322
AIIcl 88	*ΔadcAII*	Cm	New transformation	1	Normal	None
AII+Pcl4	*ΔadcAII* + *phtD*	Cm	New transformation	2.2	Thick	Not sequenced

a aa, amino acid position.

## DISCUSSION

In this work, we have described that mutation of the zinc transporter lipoprotein gene *adcAII* in the S. pneumoniae D39 strain leads to an unexpected and striking increase in capsule expression in 42% of the resulting mutants. This phenotype occurred with *ΔadcAII* mutations made by transformation either with a PCR construct or with genomic DNA from another *ΔadcAII* mutant and was stable over many bacterial generations. A similar mucoid phenotype was also observed with the *ΔadcAII* mutation in two of the four other S. pneumoniae capsular serotypes investigated. The increased capsule quantity was very marked, with EM showing a greater-than-5-fold increase in capsule width and nuclear magnetic resonance (NMR) showing a 60% increase in the quantity of monosaccharides in purified capsule. This level of increase in capsule expression is markedly greater than that seen between opaque and transparent TIGR4 capsular switched (less than 2-fold) (31) and 6B strains (32), justifying describing the D39 *ΔadcAII* strain as hyperencapsulated. The phenotypic consequence of the increased capsule expression was a high degree of resistance to complement-mediated immunity and hypervirulence in mouse models of pneumonia and sepsis. These phenotypes are exaggerated versions of the well-described effects of the capsule on S. pneumoniae evasion of host immunity (7), demonstrating that under a normal level of expression the capsule effects on immune evasion have not reached maximal potential. Previous data have shown that capsule expression comes at a metabolic cost which inhibits growth when cultured in defined medium and that the capsule prevents adhesion by respiratory epithelium (26, 33, 34). However, surprisingly, these negative aspects of capsule expression were not identified with the hyperencapsulated *ΔadcAII* strain. The serotype 2 S. pneumoniae capsule repeating unit is a hexasaccharide consisting of one glucuronic acid, two glucoses, and three rhamnoses (6, 35). NMR demonstrated that the relative proportion of glucose to rhamnose was altered in the *ΔadcAII* strain compared to WT D39, shifting from almost 1 to 1 in the latter to closer to the expected 2-to-3 ratio. This would be compatible with an increased proportion of the total S. pneumoniae glucose pool being used for capsule production. The larger comparative increase in capsule width compared to changes in monosaccharide quantity suggests the organization of the capsule may have been altered, perhaps with more loosely packed but longer capsule strands in the *ΔadcAII* strain compared to D39.

Why there is increased expression of the capsule in the *ΔadcAII* strain is not clear. The close linkage to *adcAII* suggests a role for disruption of zinc utilization, yet the hyperencapsulated phenotype did not occur with mutation of the other S. pneumoniae zinc uptake lipoprotein gene *adcA* (19) and was not affected by zinc availability. Combined deletion of *adcA* and *adcAII* was also not associated with the hyperencapsulated phenotype, but the double mutation had major effects on S. pneumoniae physiology (19) which could have obscured or suppressed the mucoid phenotype. Overall regulation of S. pneumoniae capsule expression is poorly understood and is further complicated by the large number of different capsular carbohydrate structures with potentially significant differences in regulatory mechanisms. Factors affecting thickness of the capsule layer include regulation of *cps* locus gene expression by RitR (an orphan two-component signal transduction component) (36), CpsR (a GntR family regulator) (37), and RegM (38), as well as the conserved 5′ *cpsABCD* (also termed *wzg*, *wzh*, *wzd*, and *wze*) genes of the *cps* locus (39–41). Two S. pneumoniae quorum-sensing systems (LuxS/AI-2 and the Rgg/small hydrophobic peptide system) increase capsule thickness (42–44), which can also be regulated independently of gene transcription by the supply of capsule monosaccharide precursors (45) or by increased capsule shedding mediated by LytA (12). However, our transcriptome analysis did not identify increased *cps* locus gene expression or any effects on the abovementioned known regulators of capsule expression in the *ΔadcAII* strain.

Another potential mechanism causing the hyperencapsulated phenotype in the *ΔadcAII* mutant was identified by effects on transcription of the SPD_0450-0453 locus. This encodes the SpnD39III (ST5556II) type I restriction-modification system, allelic variants of which correlate with capsule thickness for several serotypes (14–16). We found that the hyperencapsulated phenotype of *ΔadcAII* mutants was associated with a predominance of either the SpnD39IIIC or F allelic variant, whereas SpnD39IIID was the dominant allele for the majority of *ΔadcAII* mutants with normal capsule thickness. This link between the hyperencapsulated phenotype of the *ΔadcAII* strain and specific alleles of the SpnD39III (ST5556II) system seems unlikely to be coincidental given the known effects of this restriction-modification system on capsule expression. However, transformation with fixed SpnD39III (ST5556II) alleles, including those associated with normal capsule thickness (A and D), did not alter the hyperencapsulated phenotype of the *ΔadcAII* mutant, showing that any effects of SpnD39III alleles on the capsule thickness of the *ΔadcAII* mutation are not readily reversed by switching alleles. This situation is further confused by the similarity in allele composition of the wild-type D39 strain and the AIIL *ΔadcAII* mutant and by differences between our data and published papers in which SpnD39III alleles are linked to thick or thin capsule phenotypes. Manso et al. found that A, E, and F allele strains were largely opaque but C strains were more transparent, Li et al. found that only E allele strains (termed *hsdSa* in their paper) were opaque, and Oliver et al. found that the A and B alleles were opaque and the others transparent (14–16). The presumed mechanism of capsule regulation by SpnD39III is differential methylation of genes or regulatory regions (14, 15), but the genes involved remain undetermined. Our transcriptome data have identified multiple additional genes showing differential expression between hyperencapsulated *ΔadcAII* strains and wild-type D39 or a normal-capsule-width *ΔadcAII* mutant, some of which could be involved in mediating increased capsule expression. These include three operons annotated as being involved in pyrimidine metabolism, suggesting a potential role for pyrimidine in controlling capsule expression. Which genes showing differential expression between the *ΔadcAII* strains and WT D39 strains are involved in the capsular phenotype and whether differential regulation is related to differences in methylation will require considerably more detailed genetic studies.

Interestingly, 50% of independently obtained hyperencapsulated *ΔadcAII* strains contained an identical nonsynonymous SNP affecting the *cps* locus gene *csp2E*. The SNP is predicted to affect the cytoplasmic tail of Csp2E, a glucose phosphate transferase that initiates the assembly of capsule components on the cell membrane and is partially conserved among most capsular serotypes (39). Point mutations of *cps2E* that affect capsule expression have been previously described (32, 39), suggesting a causative role for this SNP for the *ΔadcAII*-related capsule phenotypes. However, the same SNP was not present in one lineage of *ΔadcAII* with increased capsule thickness (the original transformant and four back-crossed derivatives) and was also identified in one out of eight normal-capsule-thickness *ΔadcAII* strains. All the unencapsulated *ΔadcAII* transformants also contained the same SNP in *csp2E* predicted to introduce a stop codon. This high frequency of *cspE2* stop codon mutations suggests that partial deletion of *adcAII* causes significant physiology stress to S. pneumoniae that may induce loss of capsule production as an escape mutation.

To conclude, we have identified that in the S. pneumoniae D39 strain a hyperencapsulated phenotype is an unexpected consequence of targeted mutation of *adcAII*, which encodes a zinc ABC transporter lipoprotein. This strain will be a useful tool for investigating how the capsule affects S. pneumoniae interactions with the host. The hyperencapsulated phenotype partially correlated with both a nonsynonymous SNP in *cps2E* and changes in allelic dominance within the SpnD39III (ST5556II) restriction-modification system. Further investigation of genes showing differential expression between normal and hyperencapsulated D39 strains could help to further identify the underlying mechanism(s) controlling S. pneumoniae capsule thickness.

## MATERIALS AND METHODS

### Bacterial strains and growth conditions.

The Δ*adcAII*, Δ*phtD*, Δ*adcA/adcAII*, and Δ*adcAII/phtD* mutant strains were created either in the wild type or in the Δ*cpsD* D39 strain as well as in wild-type serotype 4 (TIGR4), 6A, 6B (strains 6Aa and 6Ba, respectively, from the work of Hyams et al. [31]), and 17F (46) strains by gene replacement using genomic DNA or PCR-amplified fragments obtained from the corresponding R6 mutants and standard transformation protocols for S. pneumoniae (19). The *cat* and *kana* genes were inserted in the reverse orientation without promoter or terminator sequences to avoid affecting expression of adjacent genes. Mutant identities were verified by PCR with primers flanking the cloned regions. S. pneumoniae was grown at 37°C with 5% CO_2_ in air in THY or on Columbia agar containing 5% blood. Working stocks grown to an optical density (OD) of 0.4 (∼10^8^ CFU/ml) were made using THY and stored at −80°C in 10% glycerol as single-use aliquots. CFU were confirmed by colony counting of log_10_ serial dilutions of bacteria cultured overnight on 5% Columbia blood agar. Growth curves were determined by measuring OD_595_ for bacteria cultured in 2.5 ml of THY or chemically defined medium (CDM) supplemented with 33 μM Zn in 24-well plates sealed with a transparent film and incubated at 37°C in a FLUOstar reader. To measure blood growth, 1 × 10^6^ CFU/ml of S. pneumoniae was inoculated into 1 ml of heparinized human blood and incubated at 37°C, with plating of serial dilutions at 0, 4, and 6 h to assess bacterial CFU.

### Capsule size measurement and microscopy.

An indirect method was developed to measure capsule size by determining the size of the bacterial pellet. Briefly, 12 ml of culture was centrifuged, the pellet was resuspended in 120 μl of PBS, and 35 μl was loaded in a microcapillary tube. After centrifugation for 15 min at 800 × *g*, the height of the pellet within the tube was measured with a ruler. Electron microscopy of mid-log-phase S. pneumoniae fixed in 3% paraformaldehyde (PAF) was performed using a ruthenium red and London resin capsule-preserving protocol as previously described (33). Capsule thickness was calculated by direct measurement of the surface layer for 30 randomly chosen S. pneumoniae bacteria/strain using ImageJ software.

Confocal microscopy on bacteria was performed using an Olympus FV1000 confocal laser scanning microscope with a 63× objective. Bacteria were fixed for 30 min with 4% PFA (Sigma) on slides (Thermo Scientific; SuperFrost Plus 10149870) and subsequently stained with anti-serotype 2 antibody (Statens Serum Institute) plus Alexa Fluor 546-conjugated anti-rabbit antibody. DNA was stained with 4′,6-diamidino-2-phenylindole (DAPI).

### Capsular polysaccharide extraction and quantification.

Capsular polysaccharides were extracted from 1 liter of culture, and bacteria were resuspended in 10 ml of 0.15 M Tris buffer (pH 8) supplemented with 0.1% deoxycholate and incubated for 10 min at 37°C and then for 35 min at 50°C. Cell debris was removed by centrifugation under acidic condition. Proteins were eliminated from the supernatant by two successive extractions using a 5:1 ratio of chloroform and butanol, before precipitating capsular polysaccharides in 80% ethanol. Pellets were dried, resuspended in 0.1 M phosphate buffer (pH 7.2), and incubated with DNase and RNase for 1 h at 37°C, and then trypsin was added for 2 h at 37°C before purification of capsular polysaccharide by ion exchange on a column of DEAE Sepharose. Monosaccharide composition was established by GC and GC-MS as alditol acetate derivatives. Briefly, samples were hydrolyzed in 4 M trifluoroacetic acid (TFA) for 4 h at 100°C and reduced with sodium borohydride in 0.05 M NaOH for 4 h. Reduction was stopped by dropwise addition of acetic acid until pH 6 was reached, and borate salts were codistilled by repetitive evaporation in dry methanol. Peracetylation was performed in acetic anhydride at 100°C for 2 h. All monosaccharide derivatives were identified according to their specific retention times and electron ionization MS (EI-MS) fragmentation patterns (47).

### Phagocytosis, neutrophil killing, complement deposition, and adhesion assays.

Flow cytometry phagocytosis and complement deposition assays were performed as previously described (7, 48) using S. pneumoniae incubated for 30 min with human serum (25%), human heat-inactivated serum (25%), or just Hanks balanced salt solution (HBSS) medium. For macrophage phagocytosis, THP-1 monocytes cultured in suspension in RPMI medium supplemented with 10% fetal bovine serum (FBS) were treated for 24 h with 10 nM phorbol 12-myristate 13-acetate (PMA) to induce cell adhesion and macrophage differentiation. Flow cytometry was performed using a fluorescence-activated cell sorting (FACS) Verse machine (BD), and the data were analyzed with FlowJo software. For neutrophil killing assays, fresh human neutrophils were purified using a magnetically activated cell sorting (MACS) neutrophil isolation kit (Miltenyi Biotec) and resuspended in HBSS medium at a concentration of 1 × 10^6^ cells/ml. S. pneumoniae previously incubated with human sera was incubated with the neutrophils at a multiplicity of infection (MOI) of 1:500 (bacteria to neutrophils) in a 48-well plate for 45 min at 37°C. Adhesion assays were performed on Detroit 562 human nasopharyngeal cells (ATCC CCL-138) as previously described (34, 49) using 3 × 10^5^ cells/well seeded into 24-well plates, infected with MOIs of 25 and 50, and incubated for 1 h before being washed three times with PBS, followed by addition of Dulbecco’s modified Eagle’s medium (d-MEM)-1% saponin for 10 min and plating of serial dilutions to count bacterial CFU.

### Genome sequencing and transcriptional microarray analysis.

Mutant strains were genome sequenced by the Wellcome Trust Centre for Human Genetics (Oxford, United Kingdom) using an Illumina MiSeq sequencer. Sequences were assembled using Velvet, annotated using Prokka, and mapped to the published D39 (R00000036) reference genome. Bases and single-nucleotide variants were identified using the SAMtools “mpileup” command and Bcftools. Sites were filtered to a minimum depth of five reads at each and a single-nucleotide variant quality of 25, and the Integrated Genome Viewer was used to visualize mapping and coverage. Gene transcriptome microarrays were performed as described previously (34). Briefly, RNA was extracted with the RNeasy minikit (Qiagen), and labeled cDNA was prepared using Cy3-dCTP (GE Healthcare, United Kingdom) and SuperScript II reverse transcriptase with random hexamer primers (Life Technologies). Labeled cDNA was hybridized overnight to the BμG@S SPv1.4.0 Agilent SurePrint platform (Agilent Technologies) microarray designed by the Bacterial Microarray Group at St. George’s, University of London. After hybridization, washed slides were scanned immediately, using an Agilent high-resolution microarray scanner, at 5-μm resolution; scanned images were quantified using Feature Extraction software v 10.7.3.1 (Agilent Technologies); and statistical analysis of raw intensity data was performed in GeneSpring v14.9.1 (Agilent Technologies). Data for 3 independent biological replicate experiments were analyzed and normalized using a 75th percentile shift plus baseline normalized to the median for the related control sample for each biological replicate. Statistically significant (*P* < 0.05) differences between strains were identified in an unpaired *t* test with Benjamini and Hochberg false-discovery rate correction.

### Assessing allelic variants of SpnD39III.

Primers AMRE74L (5′ 6-carboxyfluorescein [FAM] label, FAM-GGAAACTGAGATATTTCGTGGTGATGATGGGA) and AMRE59 (CCTGATCGAGCGGAAGAATATTTCTGCCGAGGTTGCC) were used to PCR amplify a 4.2-kb fragment from S. pneumoniae under the following conditions: denaturation at 95°C for 5 min, followed by 40 cycles of 1 min of denaturation at 95°C, 1 min of annealing at 68°C, and 5 min of extension at 68°C, with a final extension of 10 min at 68°C. PCR products were digested using 1 U DraI, 2 U PleI, and 1× CutSmart Buffer (all from New England Biolabs, United Kingdom) in a 20-μl volume. Each FAM-labeled SpnIII variant has a unique size that can be distinguished through capillary electrophoresis on an ABI Prism gene analyzer (Applied Biosystems, USA) and analyzed using Peak Scanner v1.0 software. Genomic DNA for transformation using locked SpnD39III (ST5556II) alleles due to an inactivated *creX* gene was obtained from preexisting strains (14).

### Animal models of infection.

All animal experiments conformed to institutional and United Kingdom Home Office guidelines and regulations. Outbred CD1 sex-matched white mice were used for infection experiments using established models of infection (50–52). For the nasopharyngeal colonization model, 10^7^ CFU of bacteria in 10 μl was administered by intranasal inoculation under light halothane general anesthesia, and nasal washes were obtained after 5 days. Mice were inoculated by intraperitoneal (i.p.) injection of 5 × 10^4^ CFU for the sepsis model and by intranasal (i.n.) inoculation under isoflurane inhalational anesthesia of 5 × 10^6^ CFU for the pneumonia model. Mice were sacrificed after 24 h (i.p.) or 48 h (i.n.), and serial dilutions of blood and lung homogenates were plated to enumerate target organ CFU. For the sepsis model, disease development was also monitored by observing mice three times a day (*n* = 8). For colonization and sepsis competitive infection models, mice were inoculated with a 50/50 ratio of D39 wild type and Δ*adcAII* strain to determine the competitive index (CI; ratio of mutant to WT strain recovered from mice divided by the ratio of mutant to WT strain in the inoculum).

### Statistical analysis.

Statistical analyses were conducted using Prism 7 (Graph Pad, USA). Parametric data are presented as means, and error bars represent standard deviations. Nonparametric date were analyzed using the Mann-Whitney U test. For the disease development model, data were analyzed using the log rank (Mantel-Cox) test.
